# Delivering bioactive cyclic peptides that target Hsp90 as prodrugs

**DOI:** 10.1080/14756366.2019.1580276

**Published:** 2019-03-01

**Authors:** Yuantao Huo, Laura K. Buckton, Jack L. Bennett, Eloise C. Smith, Frances L. Byrne, Kyle L. Hoehn, Marwa N. Rahimi, Shelli R. McAlpine

**Affiliations:** a School of Chemistry, University of New South Wales, Sydney, Australia;; b School of Biotechnology and Biomolecular Sciences, University of New South Wales, Sydney, Australia

**Keywords:** Peptide drug delivery, protein-protein interactions, prodrugs, heat shock protein 90, cell permeability

## Abstract

The most challenging issue facing peptide drug development is producing a molecule with optimal physical properties while maintaining target binding affinity. Masking peptides with protecting groups that can be removed inside the cell, produces a cell-permeable peptide, which theoretically can maintain its biological activity. Described are series of prodrugs masked using: (a) *O*-alkyl, (b) *N*-alkyl, and (c) acetyl groups, and their binding affinity for Hsp90. Alkyl moieties increased compound permeability, P_app,_ from 3.3 to 5.6, however alkyls could not be removed by liver microsomes or *in-vivo* and their presence decreased target binding affinity (IC_50_ of ≥10 µM). Thus, unlike small molecules, peptide masking groups cannot be predictably removed; their removal is related to the 3-D conformation. O-acetyl groups were cleaved but are labile, increasing challenges during synthesis. Utilising acetyl groups coupled with mono-methylated amines may decrease the polarity of a peptide, while maintaining binding affinity.

## Graphical Abstract

**Figure:**
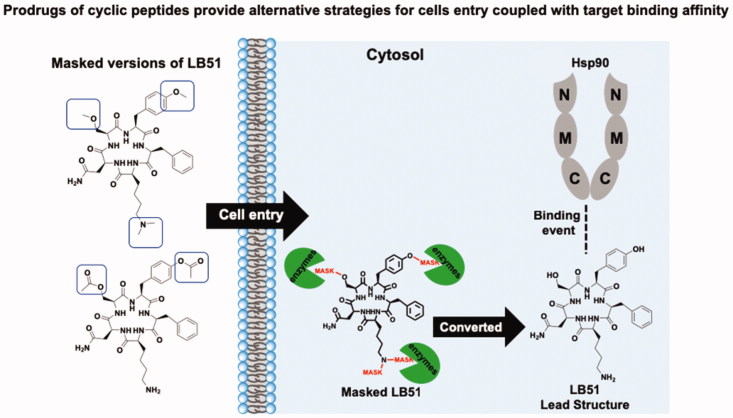

## Introduction

Peptides are evolutionarily designed by nature to inhibit protein-protein interactions (PPIs)^1–6^. Peptide scaffolds are densely packed with stereochemistry, they have rich structural diversity, and their synthesis can be accomplished in a timely manner. Peptide drugs have proven to be highly successful at regulating PPIs and drug development has increasingly relied upon the peptide field to produce new PPI modulators as therapeutics^2^ ^,^ ^7^ ^,^ ^8^. The global peptide drug market is expected to double by 2024, and reach $46.6 billion^1^ ^,^ ^8^ with ∼140 peptides being evaluated in clinical trials each year^1^.

The most challenging issue facing peptide drug development is producing a molecule with optimal physical properties while maintaining target binding affinity. The most challenging property to improve is cell permeability because structural modifications typically impact the molecule’s efficacy and aqueous solubility^9–15^. Pioneering research has been accomplished by multiple leaders in this field^12^ ^,^ ^13^ ^,^ ^16^ ^,^ ^17^ and three strategies have emerged: (1) masking polar side chains with hydrophobic moieties that can be removed within the cell, (2) incorporating d-amino acids in order to modify the conformation, and (3) *N*-methylating the backbone in order to alter the conformation and promote hydrophobicity^9^ ^,^ ^11^ ^,^ ^12^ ^,^ ^15^. Biological studies revealed that while including d-amino acids and *N*-methyl groups was favourable for membrane permeability, the irreversible chemical modifications negatively impacted the bioactivity of these molecules^9^ ^,^ ^10^. In contrast, masking peptides with protecting groups that can be removed inside the cell, produces a cell-permeable peptide, which theoretically can maintain its biological activity. Proof that a prodrug or masking strategy can succeed is evidenced by the fact that 20% of all small molecule drugs approved between 2000 and 2008 were prodrugs^18^.

**Figure 1.**
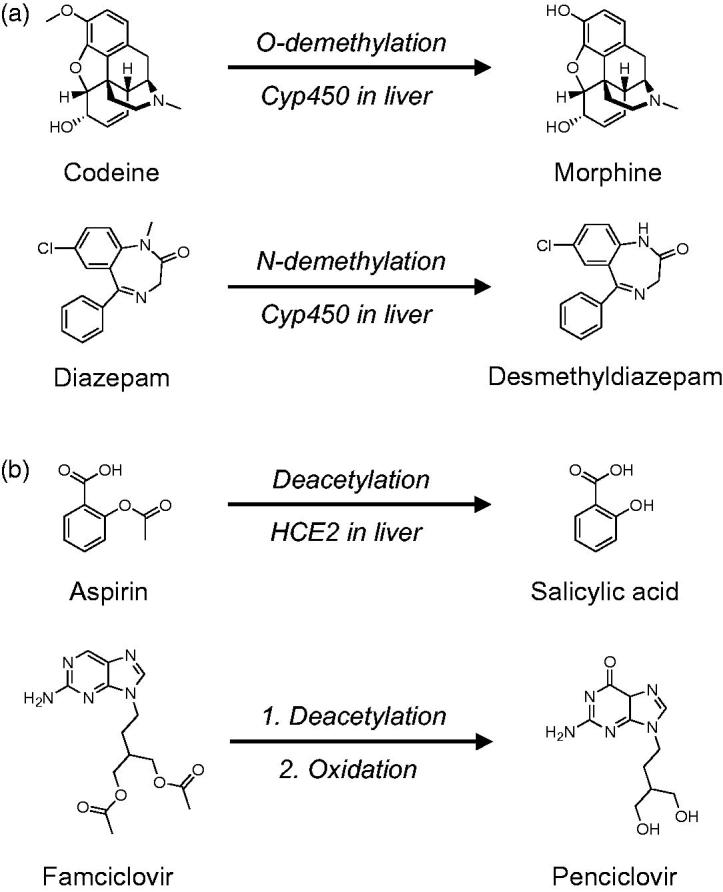
(a) *O*- and *N*-demethylation of prodrugs codeine and diazepam. (b) Deacetylation of the prodrugs aspirin and famciclovir.

Several prodrug strategies have been successfully used to produce molecules that can be unveiled upon entering the cell. *O*- and *N*-dealkylation are common metabolic processes and multiple marketed drugs, including codeine and diazepam, rely on *O*- and *N*-demethylation to produce the bioactive drug (Figure 1(a))^19–23^. Acetyl groups are also frequently used to mask alcohols, phenols, and amines in order to improve drug pharmacokinetics (Figure 1(b))^18^, where acetyl moieties are readily cleaved by ubiquitously expressed esterases^24^. Both aspirin and famciclovir have acetyl groups that are rapidly cleaved by human carboxylesterase 2 (HCE2), which yields the active drug (Figure 1(b))^25–31^.

We had previously reported molecules that targeted heat shock protein 90 (Hsp90), with the lead structures being **LB51** and **LB63** 32 ^,33^. However, these active inhibitors had poor cell permeability, and three approaches were used to make these molecules more effective at entering cells: masking groups^10^, d-amino acids^10^, and *N*-methylation^9^.

Backbone N-methylation increased the membrane permeability of **LB51**, from Papp ∼3 to between 5 and 6^9^, but this increase in permeability resulted a reduction in binding affinity. Inclusion of d-amino acids at each position of **LB51** also improved the molecule’s permeability from Papp 3 to Papp = ∼4.2–4.6^10^. However, similar to the compounds where N-methyl was added, molecules with d-amino acids all had decreased binding affinity compared to **LB51**. Although others have used the strategies described above to successfully to produce compounds with higher cell permeability, their biological activity was not assessed, or their molecules were acting on targets located outside the cell. There is no effective system that allows a peptide to enter the cell, be released, and bind effectively to its intracellular target.

Herein we describe the biological activity of molecules that were designed to target Hsp90 while maintaining cell permeability. We describe the prodrug binding affinity for Hsp90, and the ability of liver microsomes, and *in vivo* experiments to cleave three types of masking groups: (a) *O*-alkyl, (b) *N*-alkyl, and (c) acetyl. The cleavage of these side chains and the binding affinity of the final molecules are reported.

## Experimental

### General

#### AlphaLISA^®^ binding assay

The binding assays were performed using an Hsp90 (C-terminal) Inhibitor Screening Kits (cat. 50314 or 50317) purchased from BPS Bioscience. The assay was performed according to the manufacturer's protocol and utilised AlphaLISA® technology (PerkinElmer). The test compounds were dissolved in 100% DMSO and diluted with water to the desired concentration, so that the final dilution was dissolved in 5% DMSO with water. Two microlitres of the dilution was added to a 10 µL reaction so that the final concentration of DMSO was 1% in all reactions. The reactions were conducted at room temperature for 30 min in a 10 µL mixture containing assay buffer, 6 ng (24 nM) of a C-terminal Hsp90β (UniProt P08238, a.a. 527–724) or C-terminal Hsp90α (UniProt P07900, a.a. 535–732), 40 ng (60 nM) Cyp40, and the test compound. Note: for the full-length Hsp90 binding assays, C-terminal Hsp90 was replaced with 24 nM of full-length Hsp90α (Abcam cat # ab48801). After the 30 min incubation, 10 µL of buffer containing 20 µg/mL glutathione acceptor beads (Perkin Elmer) were added to the reaction mix and incubated for 30 min in the dark. Ten microlitres of 40 µg/mL streptavidin donor beads (Perkin Elmer) were then added and the final 30 µL mixture was incubated for 1 h in the dark. The AlphaLISA^®^ signal was measured using a Tecan F200 Pro multimode plate reader.

#### Metabolism assays using human liver microsomes

Human liver microsomes (Life Technologies Gibco cat. # HMMPCL) were thawed on ice and suspended in 100 mM phosphate buffer (pH 7.4) at 0.5 mg/mL. Compounds dissolved in DMSO were then added to the reaction mixture at a final concentration of 5 µM and a final DMSO concentration of 0.1%. Metabolism was initiated by adding 5 mM NADPH and the reactions were incubated at 37 °C in a water bath. Fifty microlitre aliquots of the reaction mixture were taken at various time points and added to 100 µL of cold methanol to terminate the reaction. The time points were centrifuged at 1000 × g for 5 min. Samples were stored at −20 °C until LC/MS analysis.

#### Metabolism assay in a mouse

Mice were purchased from Australian BioResources (ABR) (Moss Vale, NSW, Australia) at 7 weeks of age and shipped to a specific pathogen-free facility at UNSW Biological Resources Centre (Sydney, Australia) with 12 h light/dark cycles. Following 1 week of acclimatisation, mice were injected in the intraperitoneal space with LB55 (1.2 mg in 120 µL Milli-Q water). One hour later, mice were euthanised by CO_2_ asphyxiation and the liver was collected for drug extraction. All mouse experiments were approved by the UNSW Animal Care and Ethics Committee. To extract the compound for LC/MS analysis, the liver tissue was powdered in a mortar and pestle that had been pre-cooled with liquid nitrogen. The tissue was then homogenised in phosphate buffered saline (pH 7.4) by sonication for 2 h. The homogenate was then centrifuged at 800 × g for 10 min at 4 °C before collecting the supernatant. The drug was extracted, and the proteins were precipitated by adding 1 ml of supernatant to 9 ml of a mixture of methanol and acetonitrile (1:9 v/v). The extraction was then centrifuged at 1000 × g for 10 min at 4 °C and subsequently analysed by LC/MS.

#### Competitive binding assay

All test compounds and biotin-tagged compounds were dissolved in 100% DMSO and were diluted to a final concentration of 1% DMSO in each reaction. Each reaction consisted of C-terminal Hsp90, (100 µM), LB51-tag (100 µM), and binding buffer (100 µL). The reactions were rocked at RT for 1 h. The untagged compound was added to each reaction (0–100 µM) and incubated for 1 h. Protein pull-down was achieved by incubating each reaction with NeutrAvidin^®^ Agarose Resins (Thermo Scientific 29201 or 29204), for an hour. The NeutrAvidin^®^ Agarose Resin was pre-blocked with 1.3% of Bovine Serum Albumins (Sigma, A2153) for 1 h. Following incubation with NeutrAvidin^®^, the supernatant was removed, and the resin was washed 6 times. The beads were boiled with 5× Laemmli sample buffer, to elute the protein complex. The supernatant was loaded onto a 12% Tris-Glycine gel following the Gel electrophoresis procedure. Proteins were transferred onto a PVDF membrane and imaged following Western blotting. C-terminal Hsp90 was detected using anti-6× His-tag antibody (Abcam, ab18184, 1:1500 dilution). The relative amount of C-terminal Hsp90 bound to LB51-tag was determined, where DMSO was set to 100% binding.

#### General chemistry procedures

All chemicals were purchased from commercial suppliers (Chem-Impex International and Sigma Aldrich) and used without further purification. All moisture sensitive reactions were performed using anhydrous solvents under nitrogen gas. Removal of solvent was carried out under reduced pressure using a Buchi R-210 rotary evaporator. Samples were lyophilised on a CHRIST Alpha 1–4 LD Plus freeze dryer.

#### Solid-phase peptide synthesis

Stepwise solid-phase peptide synthesis was performed in a polypropylene solid-phase extraction cartridge fitted with a 20 µm polyethylene frit purchased from Applied Separations (Allentown, PA)

#### Resin loading

2-chlorotrityl chloride resin was weighed, transferred to the cartridge and swelled in CH_2_Cl_2_ for 30 min prior to the resin loading reaction. The appropriate Fmoc-protected amino acid was dissolved in the minimum amount of 0.4 M DIPEA in CH_2_Cl_2_. The swelled resin was then drained, and the dissolved amino acid was added, and the suspension was agitated for 4 h. The resin was then washed 3 times with CH_2_Cl_2_, 3 times with DMF, and 3 times with CH_2_Cl_2_. The resin was then dried *in vacuo* overnight. An ∼5 mg sample of resin was used to determine the amino acid loading. Twenty percent piperidine in DMF was added to the sample to cleave the Fmoc protecting group. The resin was filtered away, and the remaining solution was diluted 1 in 20 and the UV absorbance was measured at 301 nm using a Cary 50 Bio UV-Vis instrument (Sydney, Australia). DMF was used as a blank and samples were measured in a 1 ml quartz cuvette. The resin loading was then determined using the following formula:

$$Re\;\text{sin}\;\;\mathrm{loading}=\;\frac{A_{301}\times V\times d}{\varepsilon \times W\times m}$$

where *A*_301_ = optical density at 301 nm, *V* = cleavage volume (1 cm^3^), *d*=dilution factor (20), *ε* = extinction coefficient of Fmoc group (7800 ml mmol^−1 ^cm^−1^), *W*=cuvette width (1 cm), *m* = mass of resin (g).

#### Coupling reaction

Couplings were performed in DMF at a concentration of 0.3 M. Fmoc-protected amino acid (2 equiv) and HOBt (2 equiv) were mixed with the resin. DIC (4 equiv) was then added to activate the reaction. Coupling reaction was run for a minimum of 2 h while shaking (Labquake tube shaker, Thermo Fisher Scientific) at room temperature. A negative ninhydrin test was used to confirm reaction completion. Once completed, the reaction mixture was drained and the resin was subjected to Fmoc Removal. (Note: For particularly hindered coupling reactions, HOBt was replaced with HOAt.)

#### Fmoc removal

The Fmoc protecting group was removed using the following washes: DMF (3 × 1 min), 10% piperidine in DMF (2 × 1 min), DMF (2 × 1 min), *i*-PrOH (1 × 1 min), DMF (1 × 1 min), *i*-PrOH (1 × 1 min) and DMF (3 × 1 min). The resin was then ready for the next coupling reaction.

#### Cleavage

Once the desired peptide was generated, the final Fmoc protecting group was removed following Fmoc removal procedure with the following additional washes: DMF (3 × 1 min), *i*-PrOH (3 × 1 min), and MeOH (3 × 1 min). The resin-bound peptide was then dried *in vacuo* overnight. The resin was then cleaved from the linear peptide using TFE and CH_2_Cl_2_ (1:1 *v/v*) at a concentration of 10 ml/g resin. The reaction mixture was stirred at room temperature for 6 h before filtering the resin. The filtrate was concentrated and washed at least 10 times with CH_2_Cl_2_ to remove residual entrapped TFE. The product was then dried *in vacuo* overnight to produce the linear peptide.

#### Cyclisation

Macrocyclisation of the linear peptide was achieved using a cocktail of 3 coupling reagents: HATU (1 eq.), TBTU (0.8 equiv), and DMTMM (0.8 equiv). The reaction was performed in dilute conditions using anhydrous solvents at a concentration of 0.001 M. The linear peptide and coupling reagents were dissolved separately in DMF, where 20% of the final volume was used to dissolve the linear peptide and the other 80% dissolved the coupling reagents. DIPEA (4 equiv) was added to each solution. The linear peptide solution was then added drop-wise to the coupling reagents solution via a syringe pump over ∼2 h. The reaction was stirred overnight and monitored via LC/MS. Upon completion, the reaction mixture was evaporated, and the dry solid was subjected to HPLC purification.

#### Catalytic hydrogenation

Anhydrous methanol (MeOH) was prepared by purging HPLC grade MeOH with N_2_ gas over molecular sieves (3Å) for 40 min. A suspension of the protected cyclic pentapeptide and 10% Pd/C (0.1 equiv) in anhydrous MeOH (4 mM) was stirred under a H_2_ atmosphere for 2 h. Reaction progress was monitored via LC/MS. Following reaction completion, the mixture was filtered through a sintered frit and the frit was washed with MeOH.

#### HPLC

Semi-preparative HPLC for purification was performed using a GRACE VisionHT C18 column (5 µm, 22 × 150 mm) or a Phenomenex Aeris XB-C18 column (5 µm, 21.2 × 150 mm) on a Shimadzu Prominence system. The mobile phase consisted of Milli-Q water with 0.1% (v/v) formic acid (Mobile Phase A), and HPLC grade acetonitrile with 0.1% (v/v) formic acid (Mobile Phase B) at a flow rate of 5 ml/min, starting at 95% Mobile Phase A and 5% Mobile Phase B.

#### LC/MS

LC/MS analyses were performed using either a Phenomenex Aeris XB-C18 column (3.6 µm, 2.1 × 100 mm) or a Phenomenex Aeris XB-C18 column (3.6 µm, 2.1 × 50 mm) on either a Shimadzu LCMS 2020 or Shimadzu LCMS 8030. The mobile phase consisted of Milli-Q water with 0.1% (v/v) formic acid (Mobile Phase A), and HPLC grade acetonitrile with 0.1% (v/v) formic acid (Mobile Phase B) at a flow rate of 0.2 ml/min, starting at 95% Mobile Phase A and 5% Mobile Phase B.

#### NMR

^1^H and 2 D NMR spectra were obtained on Bruker Avance III 600 MHz (Sydney, Australia). All samples were dissolved in deuterium oxide (D_2_O) or deuterated dimethyl sulfoxide ((CD_3_)_2_SO). Spectra were obtained at 298 K (25 °C).

## Synthesis of LB51(Ac)_2_–Cbz

The resin-bound Fmoc-Phe was synthesised following the Resin Loading procedure using 1.68 g of 2-chlorotrityl resin (1.1 mmol, 1 equiv) and 1.44 g Fmoc-Phe-OH (3.3 mmol, 3 equiv) dissolved in 0.4 M DIPEA/CH_2_Cl_2_. After 3 h and following the Resin Loading procedure, the resin loading was found to be 0.70 mmol/g. The Fmoc group was then removed using the Fmoc Removal procedure and a positive ninhydrin test was used to confirm complete removal producing Resin-O-Phe-NH_2_. Resin-O-Phe-NH_2_ of about 1.5 g (0.7 mmol, 1 equiv) was taken forward and coupled to the four remaining amino acids (1.1 g Fmoc-Tyr(Ac)-OH (2.4 mmol, 2 equiv), 0.92 g Fmoc-Ser(Ac)-OH (2.4 mmol, 2 equiv), 0.88 g Fmoc-Asn-OH (2.4 mmol, 2 equiv), and 1.25 g Fmoc-Lys(Cbz)-OH (2.4 mmol, 2 equiv)) following the Coupling Reaction procedure using either HOBt (2.4 mmol, 2 equiv) or HOAt (2.4 mmol, 2 equiv) with DIC (4.8 mmol, 4 equiv) in DMF at 0.3 M. A negative ninhydrin test or mass spectroscopy was used to confirm each coupling reaction completion and the Fmoc group was removed following the Fmoc Removal procedure. 2.02 g of the resin was cleaved for 12 h to generate the linear pentapeptide following the Cleavage procedure using 18 ml TFE/CH_2_Cl_2_ (1:1 v/v). The resulting linear peptide was dried *in vacuo* to produce HO-Phe-Tyr(Ac)-Ser(Ac)-Asn-Lys(Cbz)-NH_2_ as a pale yellow solid (420 mg, 39%.) 200 mg of HO-Phe-Tyr(Ac)-Ser(Ac)-Asn-Lys(Cbz)-NH_2_ (0.23 mmol, 1 equiv) was cyclised 0.087 g of HATU (0.23 mmol, 1 equiv), 0.059 g of TBTU (0.19 mmol, 0.8 equiv), 0.051 g of DMTMM (0.18 mmol, 0.8 equiv), 0.24 ml of DIPEA (1.38 mmol, 8 equiv) in anhydrous DMF (173 ml, 0.001 M) following the Macrocyclisation procedure. Once complete, the reaction mixture was subjected to purification via HPLC and dried *in vacuo* to yield the cyclo-Phe-Tyr(Ac)-Ser(Ac)-Asn-Lys(Cbz) as a white solid (100 mg, 24% overall)

LC/MS (ESI) m/z: [M + H]^+^ calculated for C_43_H_52_N_7_O_12_, 858.37; found, 858.20.

^1^H NMR (600 MHz, (D_2_O): δ 8.76–7.44 (m, 7H, backbone NH & Asn NH_2_), 7.43–6.72 (m, 14H, aromatic H), 5.18–4.88 (s, 2H, Cbz CH_2_), 4.68–3.65 (m, 7H, Phe αH, Tyr αH, Ser αH, Asn αH, Lys αH, Cbz NH, Ser βCH_2_), 3.17–2.57 (m, 8H, Lys εCH_2_, Phe βCH_2_, Tyr βCH_2_, Asn βCH_2_), 2.31–2.15 (s, 3H, Tyr Ac CH_3_), 1.99–1.77 (s, 3H, Ser Ac CH_3_), 1.76–1.59 (m, 2H, Lys βCH_2_), 1.51–1.23 (m, 2H, Lys δCH_2_), 1.23–0.97 (m, 2H, Lys γCH_2_).

## Synthesis of LB63(Ac)_2_–Cbz

The resin-bound Fmoc-Ala was synthesised following the Resin Loading procedure using 1.0 g of 2-chlorotrityl resin (1.1 mmol, 1 equiv) and 1.04 g Fmoc-Ala-OH (3.3 mmol, 3 equiv) dissolved in 0.4 M DIPEA/CH_2_Cl_2_. After 5 h and following the Resin Loading procedure, the resin loading was found to be 0.6 mmol/g. The Fmoc group was then removed using the Fmoc Removal procedure and a positive ninhydrin test was used to confirm complete removal producing Resin-O-Ala-NH_2_. 1.0 g of Resin-O-Ala-NH_2_ (0.6 mmol, 1 equiv) was taken forward and coupled to the four remaining amino acids (0.67 g Fmoc-Tyr(Ac)-OH (1.5 mmol, 2.5 equiv), 0.67 g Fmoc-Ser(Ac)-OH (1.8 mmol, 3 equiv), 0.66 g Fmoc-Asn-OH (1.8 mmol, 3 equiv), and 0.91 g Fmoc-Lys(Cbz)-OH (1.8 mmol, 3 equiv)) following the Coupling Reaction procedure using either HOBt (1.8 mmol, 3 equiv) or HOAt (1.2 mmol, 2 equiv) with DIC (3.6 mmol, 6 equiv) in DMF at ∼0.3 M. A negative ninhydrin test or mass spectroscopy was used to confirm each coupling reaction completion and the Fmoc group was removed following the Fmoc Removal procedure. 1.12 g of the resin was cleaved to generate the linear pentapeptide following the Cleavage procedure using 11 ml TFE/CH_2_Cl_2_ (1:1 v/v). The resulting linear peptide was dried *in vacuo* to produce HO-Ala-Tyr(Ac)-Ser(Ac)-Asn-Lys(Cbz)-NH_2_ as a pale yellow solid (240 mg, overall 50%.), 175 mg of HO-Ala-Tyr(Ac)-Ser(Ac)-Asn-Lys(Cbz)-NH_2_ (0.30 mmol, 1 equiv) was cyclised 0.115 g of HATU (0.3 mmol, 1.0 equiv.), 0.079 g of TBTU (0.24 mmol, 0.8 equiv.), 0.066 g of DMTMM (0.24 mmol, 0.8 equiv.), 0.42 ml of DIPEA (2.43 mmol, 8.0 equivalents) in anhydrous DMF (300 ml, 0.001 M) following the Macrocyclisation procedure. Once complete, the reaction mixture was subjected to purification via HPLC and dried *in vacuo* to yield the cyclo-Ala-Tyr(Ac)-Ser(Ac)-Asn-Lys(Cbz) as a white solid (41 mg, 28% overall).

LC/MS (ESI): m/z calculated for C_37_H_47_N_7_O_12_ (M + 1)=782.33, found 782.35.

^1^H NMR (600 MHz, (CD_3_)_2_SO): δ 8.61–7.79 (b, 4H), 7.52–7.41 (s, 1H), 7.40–7.28 (m, 5H, Cbz ArH), 7.27–7.18 (d, *J* = 8.5 Hz, 2H, εTyr), 7.06–6.98 (d, *J* = 8.5 Hz, 2H, δTyr), 5.00 (s, 2H, Cbz CH_2_), 4.48–3.91 (m, 7H, Ala αH, Tyr αH, Ser αH, Asn αH, Lys αH & Ser βCH_2_), 3.11–3.03 (m, 2H, Tyr βCH_2_), 3.00–2.94 (m, 2H, Asn βCH_2_), 2.24 (s, 3H, Tyr Ac CH_3_), 1.93 (s, 3H, Ser Ac CH_3_), 1.66 (s, 2H, Lys βCH_2_) 1.46–1.34 (m, 2H, Lys δCH_2_), 1.30(d, 3H, Ala βCH_3_), 1.23(m, 2H, Lys γCH_2_).

## Results and discussion

Although other Hsp90 inhibitors have been reported to modulate the C-terminus^34–40^, **LB51** was the first compound that binds directly to the C-terminal MEEVD region of Hsp90 and effectively inhibits the binding event between Hsp90 and its co-chaperone CYP40^32^ ^,33^. The apparent permeability (P_app_×10^−6 ^cm/s) of **LB51** measured in Caco-2 assays is 3.3^10^. The agreed upon threshold for considering peptides cell permeable are those with P_app_ ≥1. However, our goal was to develop our lead molecules to be cell permeable with P_app_ of ≥5, which places them in the range of small molecules marketed drugs, where their average P_app_ is 4^41^.

**Figure 2.**
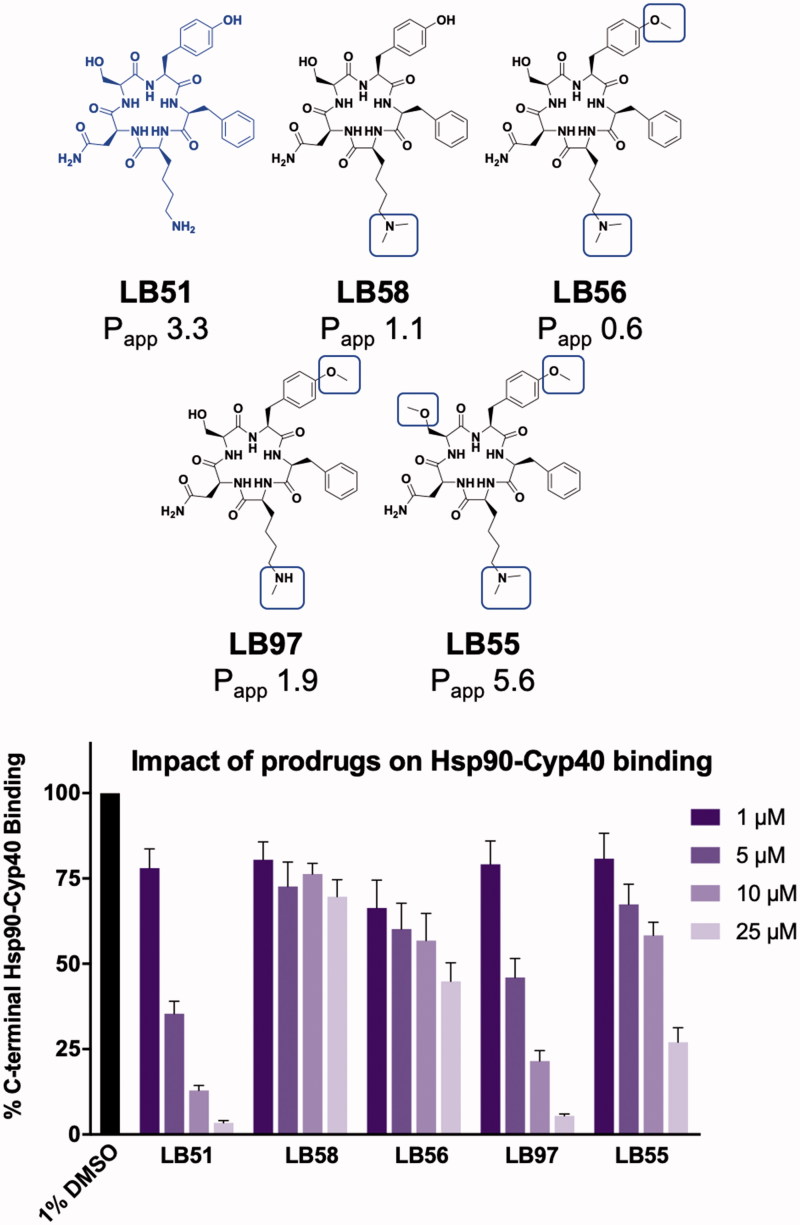
Chemical structures of molecules based on LB51 and their corresponding cell permeability and inhibitory activity in protein binding assays. P_app_ represents the cell permeability × 10^−6 ^cm/s. The graph shows the impact of series II on the Hsp90-Cyp40 interaction, compared to LB51 and DMSO. Experiments were conducted in triplicate and data represents mean ± SEM of at least 2 independent experiments.

Modifications to the side chains using the common strategy of *O*- and *N*-alkylations were the first prodrugs produced (Figure 2). Compounds **LB58**, **LB56**, **LB97**, and **LB55** were all based on the structure of **LB51** but contained polar side chains that were masked with methyl moieties (Figure 2). Compound **LB55** was the only molecule that had cell permeability P_app_ ≥5, (P_app_=5.6). However, masking groups often affect the target binding affinity, and evaluation of these molecules in binding assays was required. A biochemical assay using AlphaLISA technology was used to evaluate the efficacy of these molecules in blocking the interaction between Hsp90 and the co-chaperone, Cyp40 (Figure 2). Each compound was incubated at multiple concentrations with the C-terminal domain of Hsp90 and full-length Cyp40. Beads specific for each protein were incubated with the reaction mixture. When the two proteins are bound to each other, the beads for one protein transfer energy to the beads of the other protein, thereby producing a signal. When the binding interaction between the two proteins is inhibited, energy transfer cannot occur, which results in no signal being produced. DMSO (1%) was used as a control, where it represents 100% binding between the two proteins.

Although **LB55** was the most cell permeable, **LB51** and **LB97** were the most effective at inhibiting the binding event between Hsp90 and Cyp40. These data indicate that although the *O*- and *N*-alkylation on the three side chains (lysine, tyrosine, and serine) all facilitated cell permeability (**LB55**), target binding affinity was dramatically impacted. The data also show that although this molecule contains a free amine, **LB51** is the most effective combination of cell permeability and binding affinity. This combination of data drove us to explore how well the molecules could be dealkylated in the cell. Specifically, we evaluated whether **LB55** could be converted into **LB51** within the cell, making it ideal for prodrug development.

Given the extensive literature precedents that describe the de-alkylation of methyl groups on amino and alkoxy moieties, it was anticipated that **LB55** would be demethylated by the metabolic enzymes in liver microsomes and converted to **LB51** ^42–44^. This metabolic assay estimates drug clearance and identifies drug metabolites that are produced in the liver. Cytochrome P450 (Cyp450) proteins are located in the endoplasmic reticulum of liver cells and ∼60% of marketed drugs are metabolised by this class of enzymes^45^ ^,^ ^46^.

**Figure 3.**
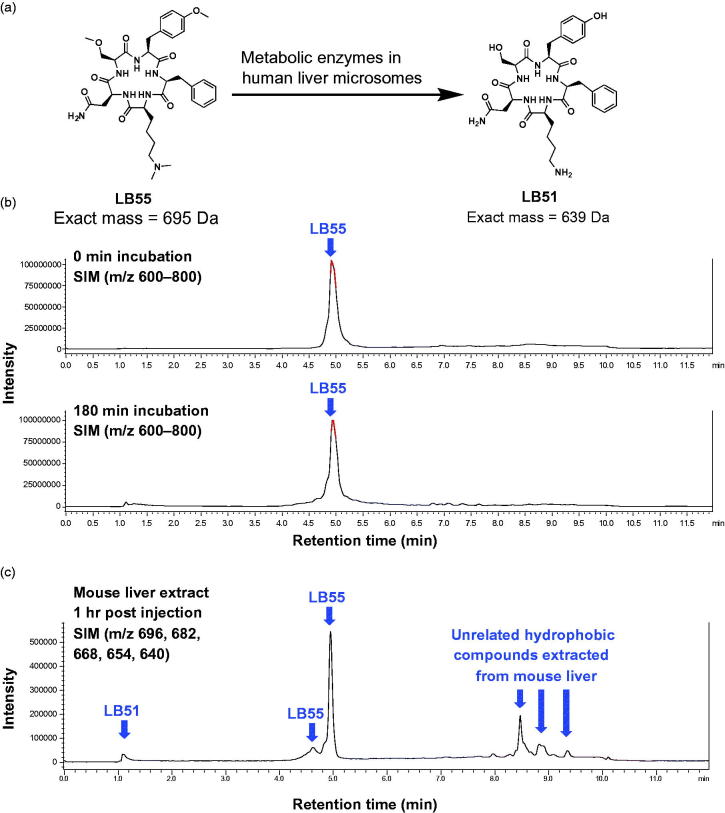
Metabolism of LB55 by human liver microsomes. (a) Structure of LB55 and LB51. (b) The LC/MS spectra of LB55 after being treated for 180 min with microsomes. LC/MS (SIM) was run scanning for all ions in the range *m/z* 600–800, which would capture all LB55 metabolites, and shows a single peak corresponding to LB55. (c) LC/MS spectrum for the mouse liver extract showing primarily parent LB55 and a small quantity of LB51 and several unrelated compounds.

Using commercially available liver microsomes that contain the full set of endoplasmic reticulum-based metabolic enzymes, including Cyp450s, in an *in vitro* system the *O*- and *N*-dealkylation processes of **LB55** were evaluated (Figure 3)^47–48^. Human liver microsomes were purchased from Life Technologies Australia Pty Ltd (catalogue number HMMCPL) and were prepared from a pool of 50 individual male and female donors. The microsomes were diluted into phosphate buffer at pH 7.4 and incubated with **LB55** at 37 °C in the presence of the enzyme cofactor NADPH, which initiates the reactions. Aliquots of the reaction mixture were taken at several time points and quenched with cold methanol, which inhibits enzyme activity. The samples were immediately centrifuged at high speed to pellet the microsomes and the supernatants were analysed via LC/MS

Isotope labelling strategies and mass spectrometry^49^ ^,^ ^50^ would have revealed any low abundance unknown metabolites with high efficiency and sensitivity. However, for simplicity and speed of assessment we opted to identify the removal of the pro-drug moieties using LC/MS in selected-ion mode (SIM). We scanned for specific mass-to-charge ratios (*m/z*) based on the molecules molecular weight. Propranolol is extensively metabolised in the liver and it produces a major metabolite (hydroxypropranolol), therefore it was used as the control compound when initially optimising the assay conditions^51^ ^,^ ^52^. After 60 min, ∼40% of propranolol was hydroxylated (Supplementary Figure S1). **LB55** was incubated with the human liver microsomes for 180 min and samples were taken at 0, 10, 20, 30, 60, 120, and 180 min and analysed via LC/MS in SIM mode. The scanning range was set at *m/z* 600–800, as this range would capture both the parent **LB55** and any demethylated metabolites including **LB51** (Figure 3).

After 180 min, the LC/MS spectra shows that no metabolism occurred of **LB55** when it was treated with liver microsomes (Figure 3(b)). Thus, despite being a common metabolic process, liver microsomes could not accomplish the de-alkylation of the methyl groups on **LB55**, suggesting that the methyl groups were inaccessible. While the microsome system provides good approximations of Cyp450-mediated drug metabolism, the commercially purchased liver microsomes have some limitations where some enzymes are present at higher concentrations in the commercial systems compared to the liver^53^ and other enzymes (especially cytosolic enzymes) are missing^53^. Thus, it is possible that some metabolic processes that would normally occur and convert **LB55** into **LB51** may still occur but could not be detected using the commercial microsomal assays. To address this issue, we conducted an *in vivo* animal study using a mouse.

The stability of **LB55** in a mouse liver was accomplished by treating the mouse with **LB55** via intraperitoneal (IP) injection at 59.4 mg/kg for 1 h. IP injections allow the drug to be rapidly absorbed through the peritoneal membrane and into the portal vein^54^. Within 60 min, drug usually accumulates in the liver, whereupon it is metabolised before it enters systemic blood circulation^55^. After 1 h of treatment, the mouse was sacrificed, and the liver was excised, snap-frozen in liquid nitrogen, and stored at −80˚C. The liver was subsequently extracted in order to isolate any parent compound and metabolites. The liver tissue was powdered in a mortar and pestle that had been pre-cooled with liquid nitrogen, and the tissue powder was homogenised by sonication in phosphate buffered saline. The homogenate was centrifuged, and the supernatant was collected. Cellular proteins were then precipitated through the addition of organic solvents (acetonitrile and methanol). The mixture was then centrifuged, and the supernatant was analysed via LC/MS in SIM mode, scanning for parent **LB55** and any demethylated metabolites including **LB51**.

The major peak from the liver extract is the starting material **LB55** (Figure 3(c)). Although a small quantity of **LB51** is produced, other metabolites are the primary products generated by mouse liver degradation. Thus, the human liver microsomes and mice liver analysis indicate that methylation was not a suitable prodrug moiety for effectively delivering **LB51** into cells and thus, a new generation of prodrugs was designed.

Since *O*- and *N*- dealkylation processes failed to cleave during standard enzyme treatments, we investigated prodrug moieties that could be removed more easily: acetyl groups are rapidly released when esterases are present. Given the failure of the methyl groups, it was important to evaluate the acetyl removal prior to synthesis of the molecules. We elected to not only test acetyl moieties but, because removing *O*- and *N*-methylations was surprisingly challenging, we also investigated trifluoroacetyl moieties. The trifluoroacetyl group is more labile than the acetyl group given the electron-withdrawing nature of –CF_3_ ^56^.

**Figure 4.**
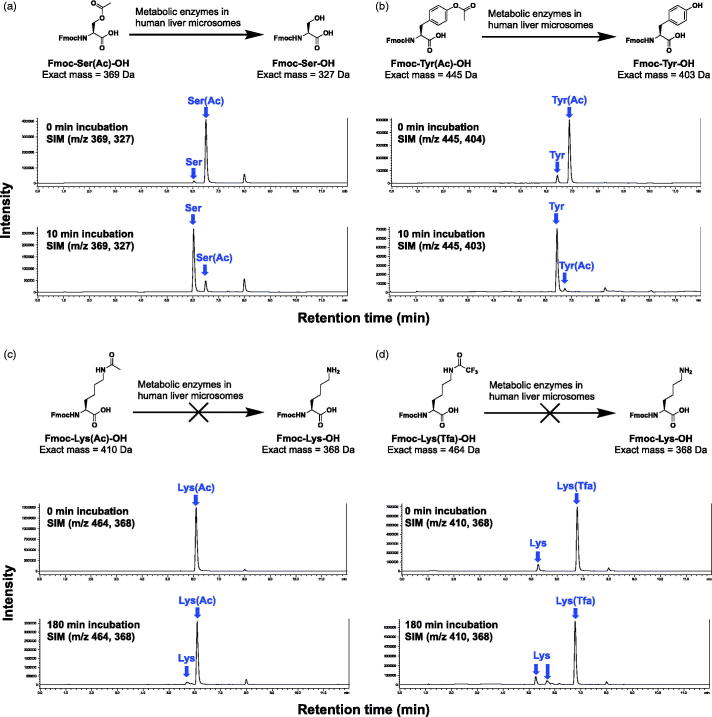
Metabolism of individual acetylated amino acids by human liver microsomes. (a) The acetyl group on serine is mostly cleaved after 10 min of being treated by liver microsomes. (b) The acetyl group on tyrosine is mostly cleaved after 10 min. (c) The acetyl group on lysine is not cleaved after 180 min incubation with liver microsomes. (d) The trifluoroacetyl group on lysine is not cleaved even at 180 min incubation with liver microsomes.

**Figure 5.**
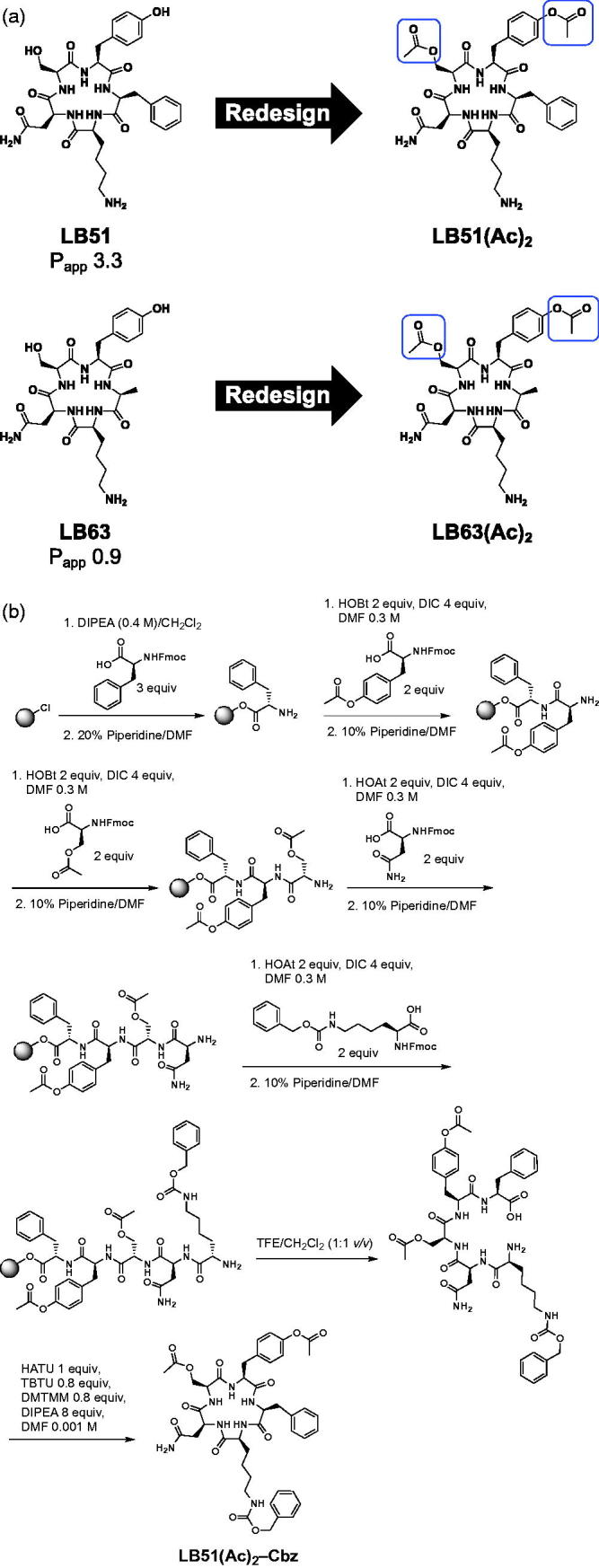
(a) Design and (b) synthesis of acetylated compounds based on compounds LB51 and LB63.

Using the same commercially available liver microsomal assay described earlier, we evaluated the removal of acetylated and trifluoroacetylated amino acids. The studies were performed on three amino acids on **LB51** that required protecting groups: serine, tyrosine, and lysine (Figure 4). Both serine and tyrosine had their acetyl groups rapidly cleaved within a 10-min incubation period. In contrast, the acetyl and trifluoroacetyl groups on lysine were stable, even after 180 min of incubation (Figure 4). The data showing that acetyl masking groups can only be removed from serine and tyrosine residues lead to the new design of prodrugs. Starting from two of our most effective compounds, **LB51** and **LB63,** we designed new prodrugs using acetylated serine and tyrosine amino acids (Figure 5(a))^32^.

Although incorporating acetyl groups is likely to improve the pharmacokinetic properties of the lead compounds, these groups are chemically labile, which makes their synthesis challenging. Acetyl groups are easily hydrolysed in both acidic and basic conditions^57^ ^,^ ^58^, and therefore standard peptide synthesis procedures needed to be modified in order to minimise acetyl hydrolysis during the synthesis of these molecules. During the synthesis of the **LB51(Ac)_2_,** the asparagine side chain was unprotected, which was also the case during the synthesis of **LB63(Ac)_2_**. The serine and tyrosine were used as purchased with acetylated groups already on the amino acid side chains. However, the amino side chain on lysine required a protecting group that could be removed under orthogonal conditions to those used for resin cleavage and leaving the acetyl moieties.

Two of the most commonly used protecting groups for lysine are tert-butyloxycarbonyl (Boc) and carboxybenzyl (Cbz)^59^. Boc is stable under basic conditions, making it most suitable to protect side chain amines when the backbone amino acid is protected using Fmoc. However, Boc is removed in acidic conditions, making it incompatible with maintaining the acetyl groups on the serine and tyrosine side chains, therefore another protecting group was used^59^. Cbz is stable under basic conditions, and it can be removed via catalytic hydrogenation, which would have no impact on the acetyl moieties^60^. Thus, Cbz was used as a protecting group on the lysine side chain (Figure 5(b)). The synthesis of **LB51(Ac)_2_** and **LB63(Ac)_2_** were produced by starting with Fmoc-phenylalanine that was loaded onto 2-chlorotrityl chloride resin. The Fmoc group was cleaved using piperidine and the resin-bound phenylalanine underwent sequential coupling and Fmoc-removal reactions with the appropriate Fmoc-protected amino acids in order to generate the resin-bound linear peptide (Figure 5(b)). The typical procedure for removing Fmoc was modified in the presence of the acetylated amino acids, and the compound’s exposure to piperidine during deprotection of the amine was reduced from 20% to 10% in order to minimise ester hydrolysis.

The linear peptide was then cleaved from the resin in mildly acidic conditions for 6 h instead of 24 h using TFE to produce the crude cleaved peptide. The cleaved peptide was then cyclised in solution under dilute conditions using anhydrous DMF, a combination of coupling reagents (HATU, TBTU, and DMTMM)^61^, and DIPEA. The reaction was monitored via LC/MS and once complete, the solvent was removed by rotary evaporation. The crude Cbz-protected cyclic peptide was then subjected to HPLC purification, which yielded the purified **LB51(Ac)_2_–Cbz** as a white solid (100 mg, 24% overall yield) (Figure 5(b)). LC/MS and 2 D NMR confirmed the structure and purity of the final product. The synthesis of **LB63(Ac)_2_–Cbz** was accomplished in a similar manner (Supp. Scheme S1) and HPLC purified to produce the compound as a white solid (41 mg, 28% yield).

Removal of the Cbz protecting group on lysine for both **LB51(Ac)_2_–Cbz** and **LB63(Ac)_2_–Cbz** was performed using catalytic hydrogenation. Catalytic palladium on carbon (Pd/C, 10% loading) was added to the compound that was dissolved in anhydrous, deoxygenated methanol. Purging the reaction flask with hydrogen gas and stirring for 2 h at room temperature under hydrogen, produced the deprotected compounds **LB51(Ac)_2_** and **LB63(Ac)_2_**, however, both molecules rapidly deacetylated after removal of the Pd catalyst (Supplementary Figure S2(a)). Several conditions were evaluated in an attempt to generate **LB51(Ac)_2_** and **LB63(Ac)_2_** including THF as an alternative solvent. However, none were successful, and we speculate that the free amine on lysine, in conjunction with trace amounts of catalytic palladium were creating conditions that removed the acetyl groups and caused decomposition.

The LC/MS immediately after reaction completion (Supplementary Figure S2(b)) shows the desired product is the major peak. Note that molecules with free lysines appear as 2 peaks on the LCMS: protonated (early retention time) and free amine (late retention time). However, after filtration and removal of the catalyst, the compound deacetylated (Supplementary Figure S2(c)). Previous studies had indicated that when the lysine of **LB51** had a single methyl masking group, it was still an effective inhibitor of the Hsp90-Cyp40 interaction. Given that the cleavage of the Cbz group was challenging, we opted to assess how effective **LB51(Ac)_2_–Cbz** and **LB63(Ac)_2_–Cbz** were when binding to Hsp90.

**Figure 6.**
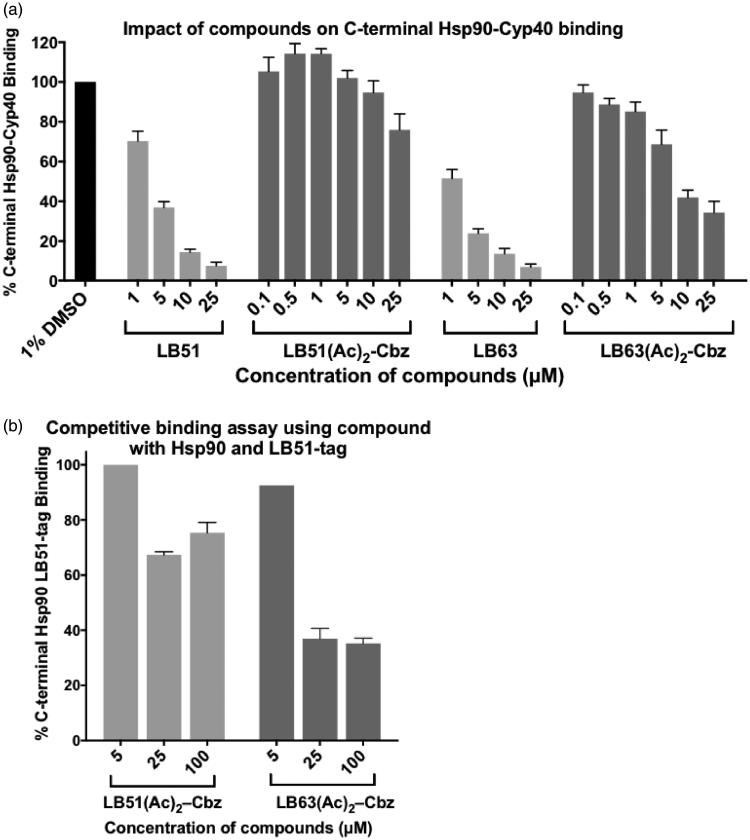
(a) The impact of LB51 and LB63 versus LB51(Ac)_2_–Cbz and LB63(Ac)_2_–Cbz on the Hsp90-Cyp40 interaction. Experiments were conducted in triplicate and data represent mean ± SEM of at least two independent experiments. (b) Competitive assays where LB51-Tag is competing for binding with LB51(Ac)_2_–Cbz, or LB63(Ac)_2_–Cbz to the C-terminal domain of Hsp90. Experiments were conducted in duplicate and data represent mean ± SEM of two independent experiments.

The alpha biochemical assay was used to evaluate the efficacy of these two molecules, **LB51(Ac)_2_–Cbz** and **LB63(Ac)_2_–Cbz,** in blocking the interaction between Hsp90 and the co-chaperone, Cyp40 (Figure 6(A)). Each compound was incubated at multiple concentrations with the C-terminal domain of Hsp90 and full-length Cyp40. DMSO (1%) was used as a negative control to represent 100% binding between the two proteins, and **LB51** and **LB63** were used as positive controls, inhibiting the binding event. **LB51(Ac)_2_–Cbz** was ineffective at inhibiting the interaction between Hsp90 and Cyp40 (Figure 6(a)). **LB63(Ac)_2_–Cbz** bound to Hsp90 with an IC_50_=10 µM, which was not as effective as **LB51** and **LB63**, which have IC_50_ values of 4 µM and 2 µM, respectively. Thus, although **LB63** was more effective than **LB63(Ac)_2_–Cbz**, the prodrug was still actively inhibiting this PPI interaction.

Competitive binding assays are useful strategies for evaluating how effectively a molecule binds to a protein target. We used this assay in order to validate the data collected in our Alpha assay and to evaluate how effectively our molecule bound to Hsp90. By incubating **LB51-biotin tag (LB51-Tag)** with Hsp90, and then adding increasing amounts of **LB51(Ac)_2_–Cbz** and **LB63(Ac)_2_–Cbz** to the reaction, we were able to assess how much Hsp90 was removed from **LB51-Tag** (Figure 6(b)). An effective inhibitor would remove a significant amount of Hsp90, and an ineffective inhibitor would not compete effectively for Hsp90 protein. Western blot analysis of how much Hsp90 remained bound to the **LB51-Tag** assessed the efficacy of the compounds. Similar to the Alpha binding assays (Figure 6(a)), the competitive binding assays using Hsp90 and **LB51-Tag** showed that **LB63(Ac)_2_–Cbz** is effective at binding to Hsp90 with an IC_50_ of ∼20 µM in this assay (Figure 6(b)). As observed in the Alpha assay, **LB51(Ac)_2_–Cbz** was not effective at binding to Hsp90 and competing off of **LB51-Tag**.

Thus, a lead molecule, **LB51**, which binds to Hsp90 and inhibits co-chaperones from interacting with Hsp90, was modified using three prodrug approaches. Our first approach involved producing cell permeable analogues of **LB51** with *O*- and *N*-methyl masking groups. However, these groups could not be removed *in vitro* with liver microsomes or *in vivo* with a mouse. Liver microsomes successfully removed the acetyl groups on serine and tyrosine side chains, however, they were unsuccessful on lysine, which led to the design of a new generation of acetylated prodrugs. However, generating these prodrugs proved to be synthetically more challenging than methylated prodrugs with *O*- and *N*-methyl moieties, given the instability of the acetyl groups.

Prodrugs of **LB51** and **LB63** with acetyl moieties on serine and tyrosine were produced. Removal of the orthogonal protecting group, Cbz, from the lysine side chain was successfully accomplished, however, the molecules rapidly degraded upon reaction completion. Given the challenges, we opted for a third approach, which was to evaluate how effectively these prodrugs that contained Cbz, **LB51(Ac)_2_–Cbz** and **LB63(Ac)_2_–Cbz,** bound to Hsp90 and blocked the interaction with Cyp40 or competed for Hsp90 versus **LB51**. **LB63(Ac)_2_–Cbz** was the most effective molecule with IC_50_ of 10 µM and ∼20 µM in the Hsp90-Cyp40 and Hsp90-**LB51-Tag** binding assay, respectively.

## Conclusions

In conclusion, our findings have implications for the development of peptide prodrugs. First, the binding affinity of a prodrug is dramatically impacted by even small changes to the peptide side chains. *O*- and *N*-alkylations on the polar side chain of **LB51** increased cell permeability from P_app_ from 3.3 to 5.6, but these compounds no longer bound effectively to Hsp90 (IC_50_  ≥ 10 µM). Molecules that maintained the acetyl moieties on serine and tyrosine were also ineffective at binding to Hsp90 with the IC_50_ > 10 µM

Thus, we conclude that traditional strategies such as alkylations used to mask polar side chains on small molecules do not work well on cyclic peptide structures. Despite dealkylation occurring in a predictable manner when liver microsomes are used on small molecules, peptide-based prodrugs that contain alkylated side chains are not metabolised under the same conditions. We believe this is because the side chains must be inaccessible to the metabolic enzymes that cleave these structures where the enzymes because of their positioning in a 3-D cyclic peptide.

We also demonstrated that using acetyl moieties as masking groups was more effective than methyl moieties because they were more easily removed from peptides. However, acetyl moieties are labile, which increases synthetic difficulty. Another conclusion that can be drawn from this study is the success of mono-methylated amines (**LB97**). The permeability of **LB97** was greater than the free lead **LB51**, while the binding affinity was similar. Thus, the optimal prodrug would involve mono-methylated amine side chains and acetylated hydroxyl side chains, where the acetyl groups would likely be removed in cells, and the methylated amine would attenuate the polarity of the amine without blocking the binding affinity. Studies examining these findings are ongoing and will be reported in due course.

## Supplementary Material

Supplemental Material
