# Supplementary material for: Delivering bioactive cyclic peptides that target Hsp90 as prodrugs
Source: J Enzyme Inhib Med Chem. 2019 Mar 1;34(1):728–39. doi: 10.1080/14756366.2019.1580276 (PMC6407599; doi:10.1080/14756366.2019.1580276)

**Delivering bioactive cyclic peptides as prodrugs**

Yuantao Huo^∞^, Laura K. Buckton,^∞^ Jack L. Bennett, Eloise C. Smith, Frances L. Byrne, Kyle L. Hoehn, Marwa N. Rahimi, and Shelli R. McAlpine*

*Corresponding author email:

Associate Professor Shelli R. McAlpine: [s.mcalpine@unsw.edu.au](mailto:s.mcalpine@unsw.edu.au)

*∞ joint first authors*

[Supplementary Figure 1 (Figure S1) 2](#_Toc525665742)

[Supplementary Scheme 1 (Scheme S1) 3](#_Toc525665743)

[Supplementary Figure 2 (Figure S2) 4](#_Toc525665744)

[LC/MS of LB51(Ac)_2_–Cbz 5](#_Toc525665762)

[^1^H NMR of LB51(Ac)_2_–Cbz 7](#_Toc525665763)

[^1^H-^13^C HSQC NMR of LB51(Ac)_2_–Cbz 8](#_Toc525665764)

[LC/MS of LB63(Ac)_2_–Cbz 9](#_Toc525665766)

[^1^H NMR of LB63(Ac)_2_–Cbz 10](#_Toc525665767)

[^1^H-^13^C HSQC NMR of LB63(Ac)_2_–Cbz 11](#_Toc525665768)

# Supplementary Figure 1 (Figure S1)

**Figure S1.** Metabolism of propranolol using human liver microsomes.

# Supplementary Scheme 1 (Scheme S1)

**Scheme S1.** Synthesis of **LB63(Ac)_2_–Cbz**, the precursor of target molecule **LB63(Ac)_2_**.

# Supplementary Figure 2 (Figure S2)

**Figure S2.** Spontaneous deacetylation of **LB51(Ac)_2_–Cbz** after workup of hydrogenation reaction.

# LC/MS of LB51(Ac)_2_–Cbz


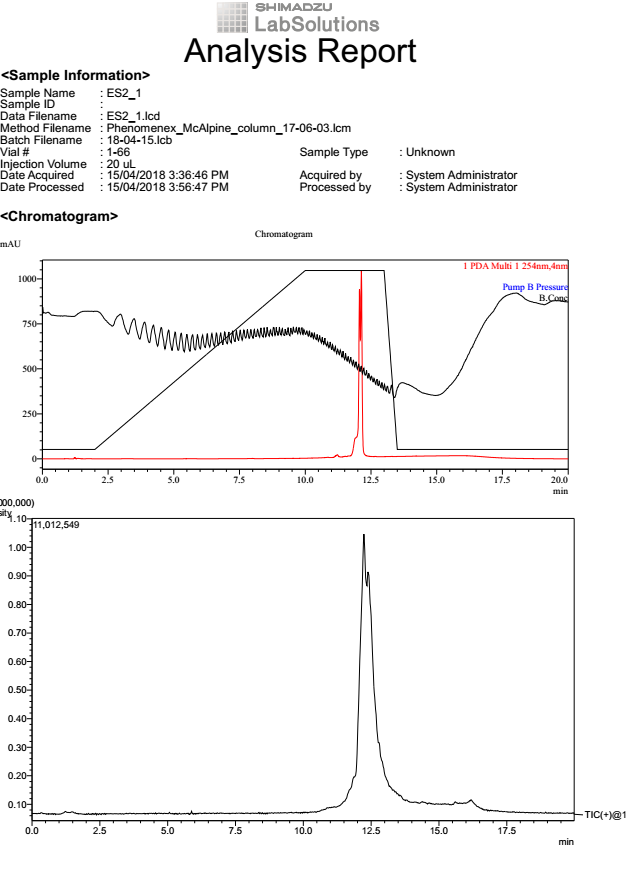


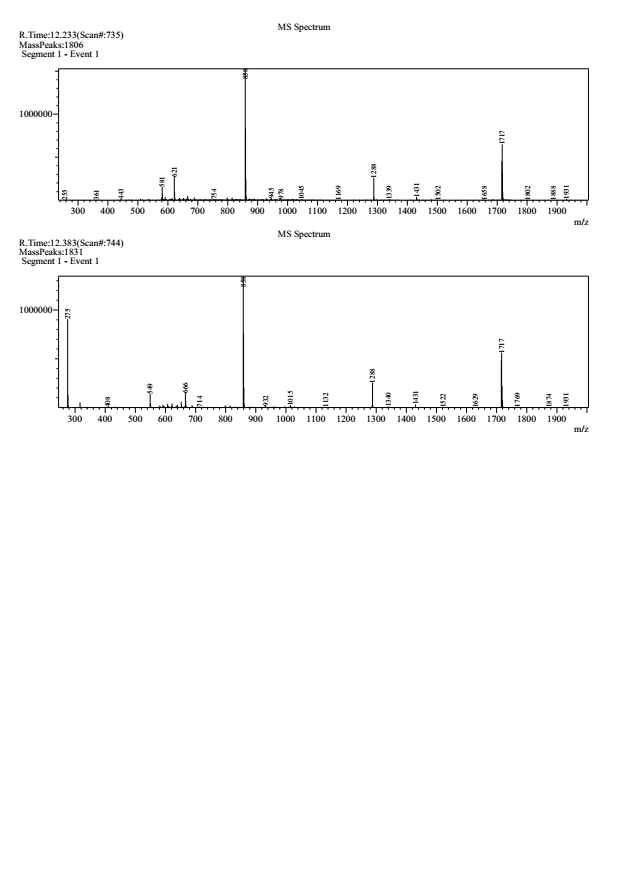


# ^1^H NMR of LB51(Ac)_2_–Cbz


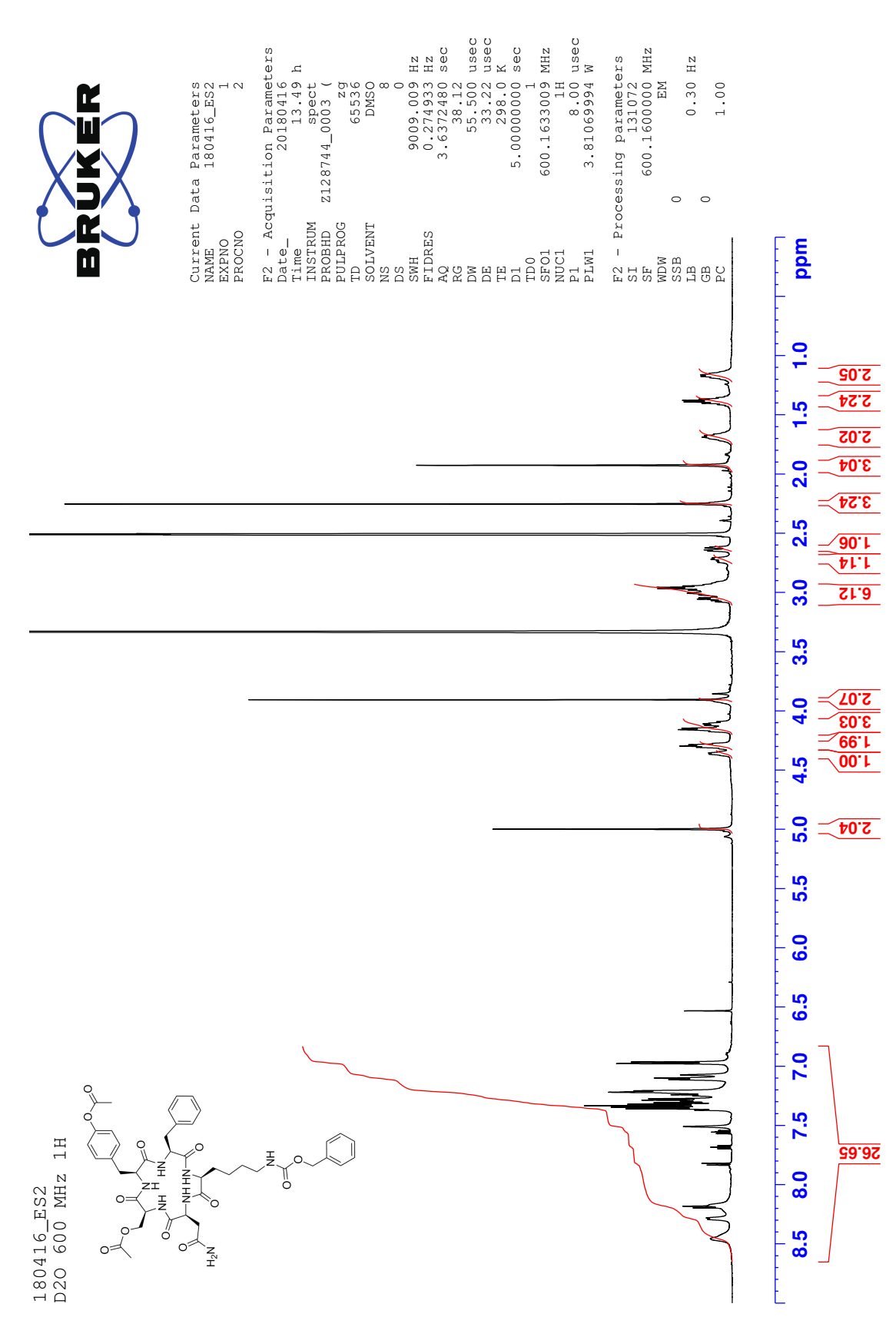


# ^1^H-^13^C HSQC NMR of LB51(Ac)_2_–Cbz


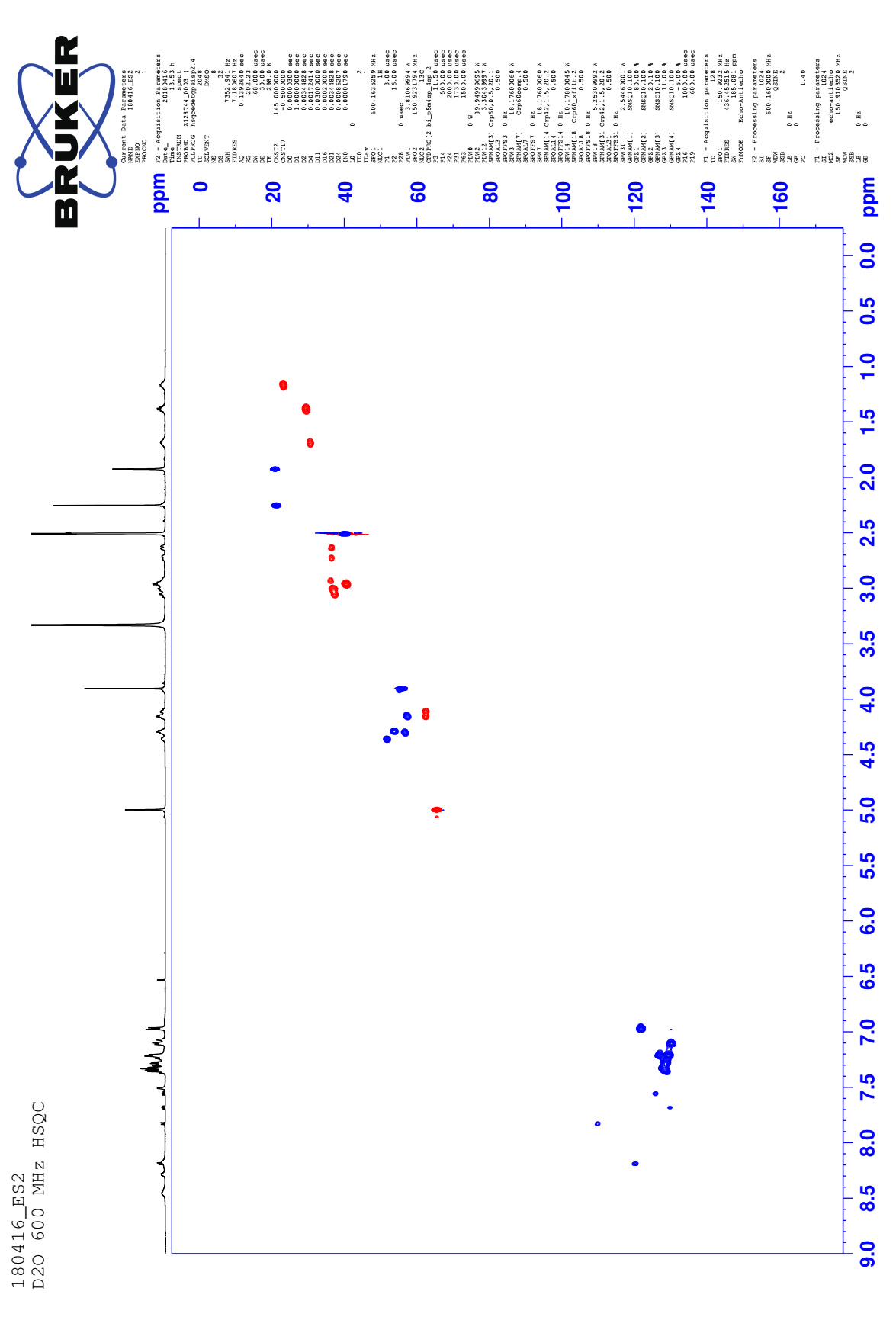


# LC/MS of LB63(Ac)_2_–Cbz

**

# ^1^H NMR of LB63(Ac)_2_–Cbz


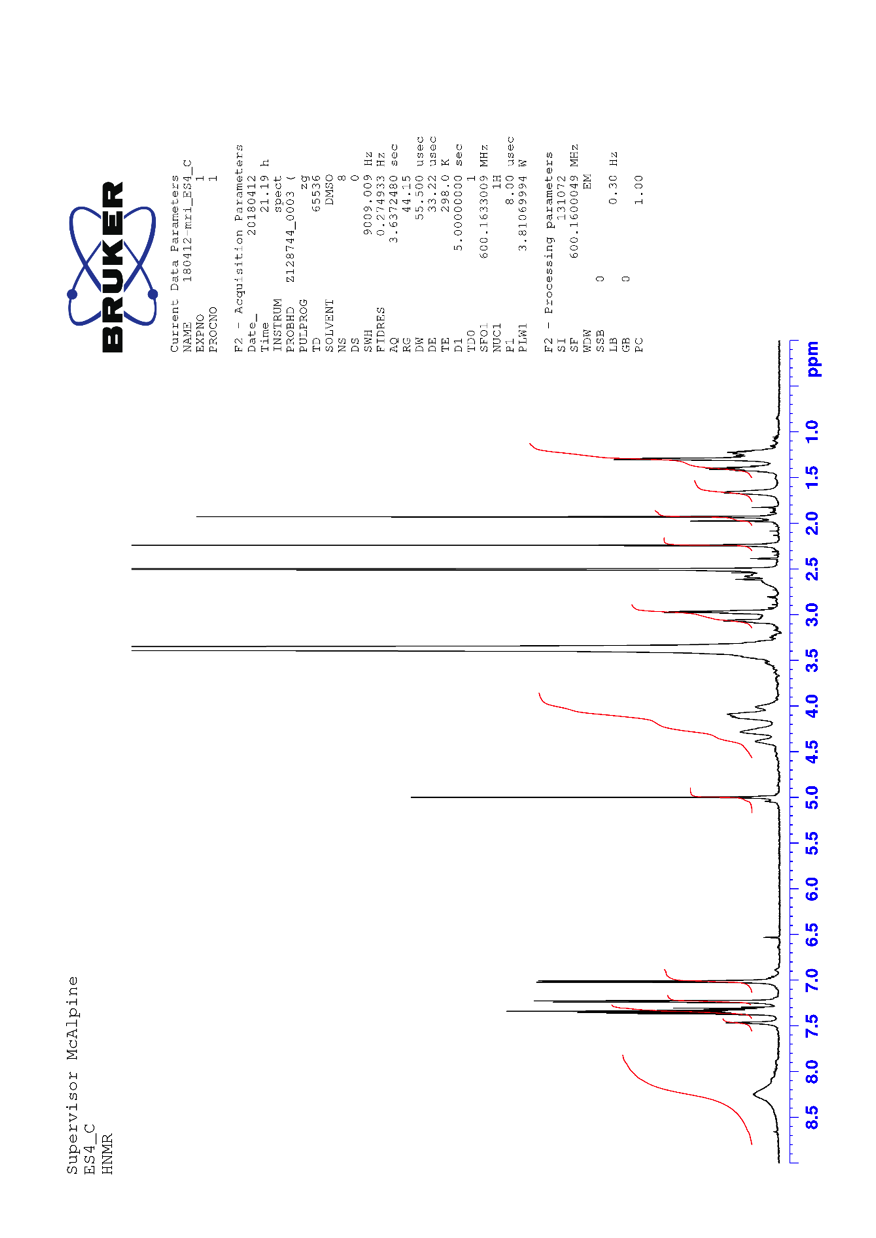


# ^1^H-^13^C HSQC NMR of LB63(Ac)_2_–Cbz


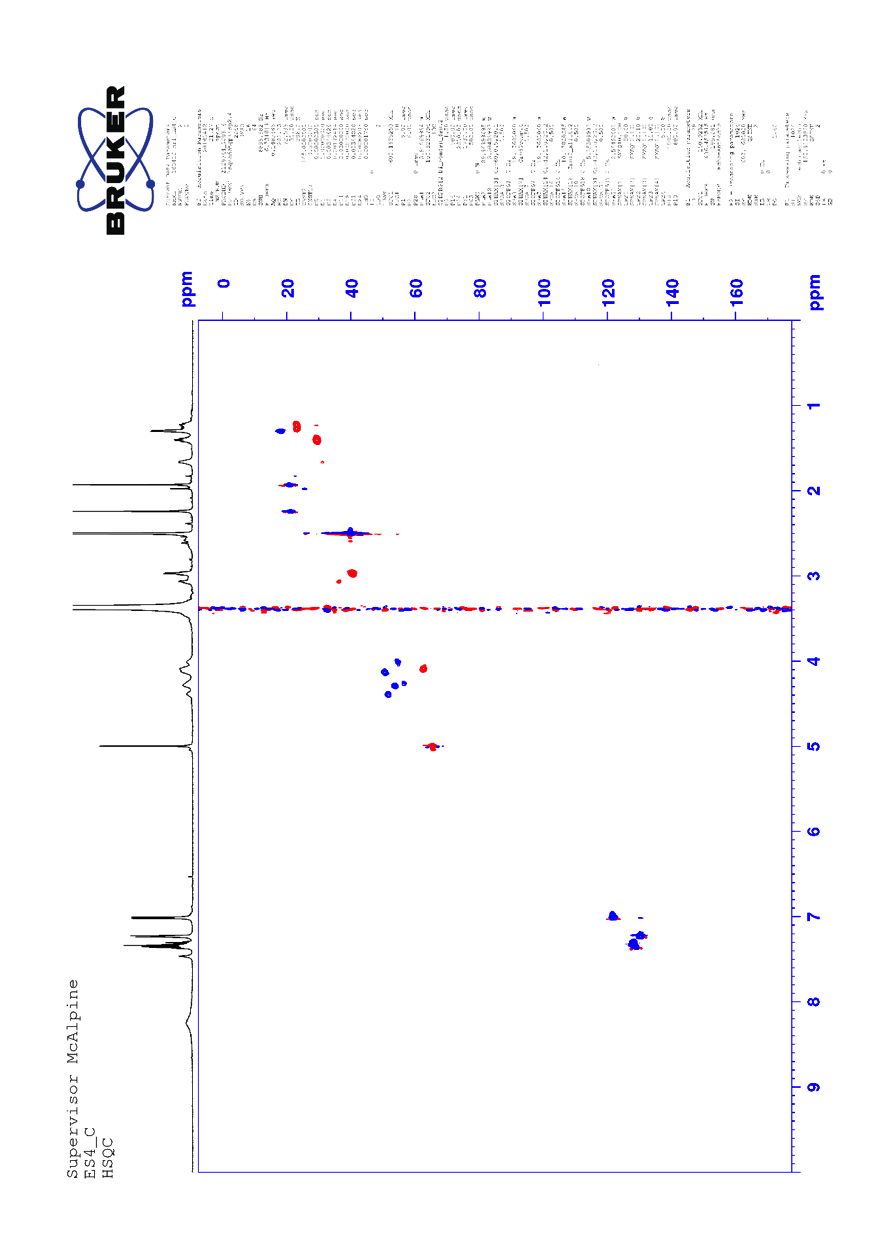

Supplement: Supplemental Material [file IENZ_A_1580276_SM2723.docx]
